# Clinical Society of the New York Polyclinical Medical School and Hospital

**Published:** 1903-08

**Authors:** 


### SOCIETY REPORTS.

### CLINICAL SOCIETY OF THE NEW YORK POLYCLINICAL MEDICAL SCHOOL AND HOSPITAL.

Meeting held April 6, 1903. The President, Dr. Alexander Lyle, in the chair.

Dr. D. S. Dougherty presented a case of paralysis of the left vocal cord, with partial paralysis of the right vocal cord. The entire larynx of the patient was congested and infiltrated, and there seemed to be no lifting of the arytenoid cartilages on the laryngeal plane. The speaker said that the patient had been operated on last November for the removal of a thyroid enlargement, and that immediately after the operation he lost his voice. It was noticed after the operation that the larynx was pushed to the left side. He returned to the hospital, and an operation was performed on the opposite side on February 10. Twelve days later he was seized with spasms of the anterior muscles of the forearm and calves of the legs, accompanied by intense pain and hyperesthesia, and on March 26 had an epileptic form of convulsion. This was the only convulsion he had had. At the present time the patient was able only to make imperfect sounds, but once or twice his voice had been quite distinct. The speaker thought that the condition was due to severance of the recurrent laryngeal nerve near the trachea, and slightly in front and to the left of the right vocal cord. The surgeon who had performed the operation thought the condition was probably due to a tetanoid infection.

Dr. W. H. Luckett, in opening the discussion, said that when the thyroid gland has been removed this condition is a common sequel and is not due to infection with the tetanus bacillus.

Dr. J. A. Bodine asked whether the patient had been operated on under general anesthesia, and was informed that ether had been used. He said that it is wrong to employ general anesthesia for operations on a goitre, as a weak solution of cocain can be used and the surgeon can see what he is doing, and thus avoid severing the nerve.

Dr. W. B. Pritchard asked if the doctor positively excluded post-operative hysteria. He thought that as the man had regained the use of his voice once or twice, the sensory disturbances, together with the tetanic condition, suggested post-operative hysteria. He thought that if organic paralysis existed his lack of phonetic powers would be lessened.

Dr. Dougherty said that he did not think that the recurrent laryngeal nerve had been severed entirely, because it would be impossible for the patient to recover his vocal powers as much as he had. He said that the post-operative hysterical element had been eliminated because the paralysis was unilateral. In post-operative hysteria, the paralysis is always bilateral, and the cords remain entirely without motion. In the case under discussion the right cord would abduct, but the left cord remained without motion except from the motion in the entire larynx.

Dr. Pritchard said that hysteria coexists with unilateral paralysis in other parts of the body, and he did not see why it could not be possible in the larynx.

Dr. Dougherty closed the discussion by saying that Dr. F. J. Quinlan had examined the patient and had found nothing to indicate hysteria.

### GUMMATOUS TUMOR OF THE LEG TREATED BY INTRAMUSCULAR INJECTIONS OF SALICYLATE OF MERCURY.

Dr. D. A. Sinclair showed a patient suffering from a gummatous tumor of the leg about the size of a silver dollar, situated in the lower third of the tibia. The history of the case was as follows: Widow, thirty-nine years old. No previous history of syphilis could be elicited. Tubercular history negative. Fifteen months ago the patient noticed a swelling of the right leg over the lower third of the tibia. This was treated by poulticing and it broke. Later, she was anesthetized by ether on two occasions and the swelling opened and the bone scraped. At the end of fifteen months the patient was worse than at any time since the growth made its appearance. She complained of severe pain and exquisite tenderness and of nocturnal pain and insomnia. In spite of the fact that no history of syphilis had been obtained, to the speaker the swelling was characteristic of gumma. On March 24 the patient received an intramuscular injection of salicylate of mercury, and a second injection of the same amount was given on April 3. The tumor rapidly diminished and at the present time is level with the skin. The discharge is very slight and pain and tenderness have entirely disappeared.

Dr. A. R. Robinson opened the discussion of this case by asking what Dr. Sinclair's experience had been with the mercurial injection alone in pure gummatous infection. The speaker thought that traumatisms might correspond with the case before the Society. There are many instances of lesions known as syphilitic in which the patient had been afflicted with syphilis some twenty years before and internal treatment with mercury alone had cured them when other measures had failed. He wanted to know whether Dr. Sinclair regarded the lesion as purely gummatous or as an inflammatory process resulting from the person having had syphilis. He said that iodide of potassium is necessary in all cases in which there is a pure gummatous lesion.

Dr. Luckett said that he thought it impossible to treat these patients with mercury alone, just as it is impossible to treat tuberculosis satisfactorily with creosote only. He thought the open-air treatment, stimulation and proper nutrition are what these patients require. He thought this patient would be greatly benefited by prolonged hot baths, producing a slight diminution of hyperemia, just enough to somewhat retard the venous supply.

Dr. Sinclair closed the discussion by saying that he regarded the case as a pure gummatous lesion.

### SEPTIC UTERUS, STRICTURE OF THE INTESTINE, INFLAMED APPENDIX.

These specimens were shown by Dr. Luckett. (1) The septic uterus contained multiple abscesses. (2) An acute stricture of the intestine was interesting because of its history. The patient, a female, aged twenty-eight years, had suffered from chronic constipation, but otherwise had never been ill. Twenty-six hours before her death she was seized with acute vomiting, spasms, and all the symptoms of acute intestinal obstruction. She was removed to the hospital, but her condition was moribund and an operation was deemed impossible. She died four hours later. At the autopsy the small intestine was removed five inches from the ileocecal valve and appeared carcinomatous to the naked eye. Specimens similar to this one have been sent to a pathologist and have been diagnosed as epithelioma, but two pathologists have pronounced this specimen to be simply an acute inflammatory growth with no sign of a neoplasm or of chronic inflammation. (3) A specimen of an appendix was presented to illustrate the almost facultative power of the omentum to carry itself from one part of the abdominal cavity to another for whatever use it is intended. If an incision is made into the abdominal cavity, even if not at the lowest end of the omentum, the border will crawl upward and try to fill the opening. This appendix was rather short and was almost on the point of perforation. It had been grasped by the omentum, and there was absolutely no adhesion to either the intestine or to the parietal peritoneum.

The paper of the evening, by Drs. Roos and Packard, entitled "The Transmissibility of Tuberculosis," was read by the latter. He said, in part:

Statistics show that one seventh of the deaths during a given year are due to tuberculosis. It is conservatively estimated that 150,000 persons die from some form of the disease in the United States annually. While certain questions involving the etiology of the disease have been settled, there is still great uncertainty as to its hereditary transmissibility. Many authorities claim that it is non-transmissible, but that predisposition is always present in a child born of tuberculous parents. Statistics and animal experience to the contrary, it can be proved conclusively that hereditary transmissibility is not only a possibility but a reality. Cohnheim was the first to suggest the possibility of direct transmission to the embryo. In substantiation of his theory, the following case was referred to: A tuberculous mother, twenty-three years of age, died in the seventh month of her first pregnancy. Directly after death the fetus was extracted by Cæsarean section. Examination of the blood from the umbilical vein, as well as of the liver, spleen and kidneys of the child, demonstrated positively the presence of tubercular deposits and of tubercle bacilli.

Lehman's case was that of tuberculosis of the placenta of a twenty-six-year-old mother, who died of general tuberculosis. Sections of the chorion showed typical miliary tubercles, and microscopical examination disclosed the presence of the bacilli. Many other cases are on record. In the experimental work of Bar and Renon they have been able to infect guinea-pigs with tuberculosis with blood taken from the umbilical vein of a fetus whose mother had been phthisical. Monti, in his lecture, was wont to say that a fetus is always prone to tuberculous infection, but that if the placenta is healthy, it usually, but with many exceptions, acts as a filter to the micro-organisms, and the child escapes.

Distinguished authorities at the present time assert that direct transmission through the sperm is a very remote possibility. Among these is Virchow, who claims that germinative infection is impossible, as the presence of the bacillus must necessarily interfere with the development of the ovum. On the other hand, in Monti's work on spermatic infection, he found that the semen of tuberculous patients, when injected into the peritoneal cavity of guinea-pigs, produced a general tuberculosis. Baumgarten and Spano have demonstrated that the seminiferous fluid of tubercular subjects whose genital organs were entirely free from pathological lesions contained numberless tubercle bacilli.

The time will come when the teaching regarding the transmissibility of tuberculosis will undergo a radical change, and authorities will then point out to the public the danger as well as the criminality of marriage between tuberculous individuals.

Dr. F. J. Quinlan opened the discussion which followed the reading of the paper. He said that he thought the ideas set forth were reasonable and possible. The soil is bad in tuberculous patients, and certainly the fruit that it puts forth is apt to have tissues below the normal resistance. Many offsprings from tuberculous patients are seen in the clinics, and their lymphatics are prone to disease germs and show evidences of the ravages of disease to such an extent as to almost compel a belief in the heredity of tuberculosis. He thought that if syphilis could spring forth anew and bear new seed in offspring of parents who had acquired this disease he did not see why tuberculosis should not act likewise. A great deal might be done to restore these children to health by clear oxygenation.

Dr. E. L. Keyes, Jr., said that his belief concerning the heredity of tuberculosis had been directly contrary to that expressed in the paper, but the cases quoted were so strongly confirmatory of direct transmission of tubercular germs from parent to offspring that he hesitated to continue his disbelief in this theory. While some of the cases were very suggestive, it was possible, nevertheless, to find one loophole of escape. The most convincing tests were upon children who were the offsprings of mothers in whom the tubercular germs were present to such an ovewhelming degree that the mother died from tuberculosis during or at the expiration of her term of pregnancy. He asked whether Dr. Packard considered these cases of direct transmission of a frequency sufficient to form a matter of clinical importance. He had a vague recollection of having read of some experiments regarding the migration of bacteria in the urinary tract. The writer made a number of experiments in which he placed germs at the meatus urinarius, and several hours afterward killed the animals and examined the kidneys, the testicles and various parts of the urinary apparatus. He kept several animals for control. He found that even tubercular bacilli could be found to have traveled up to the kidneys and up the spermatic cord into the testicles, in every way going against the stream, as it were, and yet no actual tubercular inflammation occurred. The urinary tract is regarded as the most aseptic part of the body, but he thought that it is fairly full of germs, and fancied that tubercular bacilli were often carried through the kidneys without doing any harm.

Dr. W. B. Prichard said that the attitude of the insurance companies, both here and abroad, regarding tuberculosis is of interest. Without one exception all applicants who give a tubercular history on either side are barred for a certain length of time, if not altogether, and when accepted have to pay heavier premiums than are ordinarily asked. This seems to point to the conclusion that they accept the doctrine of hereditary tuberculosis.

Dr. Packard closed the discussion by saying that the number of children of one, two and three months of age that have tuberculosis is almost conclusive proof that hereditary tuberculosis is more common than one would think. When it is considered that it is not necessary to have a tubercular genito-urinary tract in order to have tubercle bacilli in the semen, one can easily understand with little difficulty how an impregnated ovum may be tubercular. In answer to Dr. Luckett, he stated that several cases have been reported in literature which have been similar to that of Dr. Mandelbaum, of a tubercle bacillus embedded in the head of a spermatazoon. He also claimed that tubercle bacillus could be cultivated from blood, as he had been able to demonstrate cultures taken from the umbilical and portal veins.

---

### PRACTICAL POINTS ON PESSARIES.

Proper treatment of retrodeviations of the uterus with pessaries is a very important and, at the same time, one of the most difficult chapters of gynecology. The physician must know how to select the proper case for the pessary and how to select and fit the pessary to the case. When the pessary is in position, certain precautions must be taken. The vaginal wall should never be tense. The lower bar of the pessary must lie just behind the symphysis, invisible at the vulva. It should be somewhat movable up and down and free from discomfort during coitus. The wearer should not at any time feel conscious of its presence. It must be removed at the end of the first week and the vagina examined for pressure points or any signs of irritation; it should then be replaced, and again removed at least every four weeks, cleaned, polished with a dry towel and reinserted. The wearer must use daily a warm douche, to which may be added a tablespoonful of common salt or two teaspoonfuls of sodium bicarbonate. It is always wise to inform the patient that a pessary will but rarely cure uterine displacement, but will afford symptomatic relief as long as it is worn and properly cared for. Pessaries ought never to be used when there is present a purulent vaginitis, fixation of the uterus, ovaries or tubes, pus tubes or pelvic enlargements.—*A. E. Gallant, in Phil. Med. Journal, May 2, 1903.*

